# Association between CYP3A4 gene rs4646437 polymorphism and the risk of hypertension in Chinese population: a case–control study

**DOI:** 10.1042/BSR20190296

**Published:** 2019-04-17

**Authors:** Juan Wang, Hongliang Ji, Helei Jia, Dongsheng Guan

**Affiliations:** 1 The Second Clinical Medical College, Henan University of Traditional Chinese Medicine, Zhengzhou, Henan Province 450046, China; 2 Department of Endocrine and Cardiovascular, People’s Hospital of Huiji District, Zhengzhou, Henan Province 450044, China; 3 Department of Emergency, Henan Province Hospital of Traditional Chinese Medicine, Zhengzhou, Henan Province 450002, China

**Keywords:** CYP3A4, gene polymorphism, Hardy-Weinberg, knee osteoarthritis

## Abstract

Using a case–control design, we assessed the association between single nucleotide polymorphisms of *CYP3A4* gene rs4646437 polymorphism and the risk of hypertension in Chinese population. We recruited 450 hypertension patients from The First Clinical College, Henan University of Chinese Medicine between June 2017 and May 2018. There was a significant difference in genotype distribution between case group and control group (*χ^2^*=18.169, *P*=0.000). The minor A allele was significantly higher in the case group than that in the control group (31.0 vs 24.8%, *P*=0.000, odds ratio *[OR]*=1.36, 95% confidence interval [95% CI]: 1.12–1.66). Significant differences were also observed in other gene models: the GA/AA genotype did not increase the risk of hypertension compared with GG genotype (*OR*=1.16, 95% CI: 0.90–1.49, *P*=0.259). Compared with GG/GA genotype, the AA genotype also increased the risk of hypertension (*OR*=2.34, 95% CI: 1.56–3.50, *P*=0.000). For additive model, the AA genotype was significantly associated with GG genotype (*OR*=2.25, 95% CI: 1.49–3.42, *P*=0.000). The same results were found for AA vs GA (*OR*=2.50, 95% CI: 1.60–3.89, *P*=0.000). For the allele genotype, the A allele frequency was significantly higher in the case group than that in the control group (31.0 vs 24.8%, *P*=0.002). The A allele of *CYP3A4* rs4646437 was associated with an increased risk for hypertension (*OR*=1.36, 95% CI: 1.12–1.66, *P*=0.002). Our results revealed a possible genetic association between *CYP3A4* gene rs4646437 and hypertension, and the AA genotype of rs4646437 increased the risk of hypertension in Chinese Han population, and this effect could be confirmed by multivariable analyses.

## Introduction

Essential hypertension (EH), as a chronic noncommunicable disease, is also a chronic disease with high prevalence, high disability rate, and severe disease burden around the world [1]. Serious consumption of medical and social resources leads to a heavy burden for families and the society, and has become an important public health problem, especially in developing countries [2,3]. An analysis using data from the 2002 China National Nutrition and Health Survey indicated that the prevalence of hypertension was 20% among men and 17% among women. The result from the same survey showed that the figure was high (20.5%) even in the southern region of China, where the economy was comparable with that of developed countries. Therefore, it is estimated that 153 million Chinese adults were hypertensive in 2002. It is estimated that hypertensive patients contribute to 2.33 million cardiovascular deaths in China [4]. Hypertension becomes the first risk factor for cardiovascular diseases burden [5,6]. More than 2 million premature deaths are caused by elevated blood pressure every year, and direct medical expenses amount to 36.6 billion annually [7]. According to the results of a cohort study published in JAMA in China in 2016, the compliance rate of hypertension patients after treatment in China was 29.6% [8]. It has become an important subject to explore the etiology of EH. Hypertension can be divided into EH and secondary hypertension. EH accounted for 95% of all hypertensive patients [9]. Both genetic and environmental factors contribute to the pathogenesis of EH [10,11]. In recent years, genes and their polymorphisms have been found to be associated with blood pressure and susceptibility of hypertension. A large number of studies have been conducted to identify susceptibility loci in humans of hypertension [12–14]. Nonetheless, no genetic polymorphism has been identified to show consistent association with hypertension in humans.

Human cytochrome P450 (CYP) 3A subfamily members (mainly CYP3A4 and CYP3A5), a group of ferroheme enzymes, have oxidation function and play an important role in the metabolism of many endogenous and exogenous compounds [15]. The subfamily of human liver CYP mainly includes CYP1, CYP2, CYP3, and CYP4 [16]. The variation of CYP activity in individuals may be related to genetic and environment factors, mainly affected by genetic factors. CYP3A4 is the most abundant metabolic enzyme in human liver, regulating more than 50% drug metabolism [17]. The genetic sequence of CYP3A4 is also relatively conservative and few single-nucleotide polymorphisms (SNPs) were discovered to affect the activity of enzyme [18]. Recently, previous study reported that the A-allele of *CYP3A4* rs4646437 was associated with an increased risk for hypertension (odds ratio *[OR]*=2.4, 95% confidence interval *[95% CI]*: 1.10–5.20, *P*=0.021) in metastatic renal cell carcinoma patients treated with sunitinib [19]. However, there are few studies about the relationship between *CYP3A4* gene polymorphism and EH. The present study investigates the association between single nucleotide polymorphisms of *CYP3A4*/ rs4646437 gene and hypertension in the Chinese Han population.

## Materials and methods

### Study population

Using a case–control design, we recruited 450 hypertension patients from The First Clinical College, Henan University of Chinese Medicine between July 2017 and March 2018. We selected 520 health controls without the history of hypertension from physical examination center during the same period. The diagnostic of hypertension is clear: systolic blood pressure ≥140 mmHg and/or diastolic blood pressure ≥90 mmHg without antihypertension medications or antihypertension medications were used [20]. Patients with a history of hypertension, severe cardiovascular diseases, severe liver and kidney dysfunction, malignant tumor, and other autoimmune diseases were excluded from the study. We used Quanto 1.2.4 to calculate the sample size, followed by the conditions: *α*=0.05, *β*=0.10, expected *OR*=2.0, the calculated sample size is 400 in the case group and control group, respectively. The present sample size is enough. This study was approved by the institutional review board of Henan University of Chinese Medicine. Written informed consent was received from all study subjects. The research has been carried out in accordance with the World Medical Association Declaration of Helsinki, and that all subjects provided written informed consent.

### Clinical data collection

We used a standard questionnaire to collect the clinical characteristics of the study population. The collected information included age, gender, history of smoking and alcohol consumption, height and weight of body mass index (BMI) calculation, exercise time/week (none, once, twice, and more than three times), salt intake (low, moderate, and high), education level (no or primary, middle, high, and college), marriage status (unmarried, married, and others), and region (rural and urban). The BMI was calculated by weight (kg) divided by height (m) square.

Hematologic testing was conducted on the Beckman Coulter LH-750 Hematology Analyzer (Beckman Coulter, Inc., Fullerton, CA, U.S.A.), automated hematology analyzer, which measures hemoglobin (Hb) photometrically. Blood routine indices including red blood cell (RBC) count, white blood cell (WBC) count, platelet count (Plt), Hb, red cell distribution width (RDW), and mean corpuscular volume were tested using an automated biochemical analyzer. Serum creatinine level, fasting blood glucose, serum lipid status (total cholesterol [TC], low- and high-density lipoprotein [HDL-C and LDL-C], and triglyceride [TG]), ALT, and aspartate amino transferase (AST) were also determined. Serum creatinine and blood urea nitrogen (BUN) were measured on a Roche/Hitachi Modular System P (Roche Diagnostics GmbH, Mannheim, Germany) by creatinine Jaffe´, rate blanked and compensated assay. The eGFR was calculated from the calculation formula defined: 186 × SCr^−1.154^ × age in years^−0.203^ × 1.210 (if black) × 0.742 (if female), the definition of chronic kidney disease (low GFR) was an *eGFR*<60 ml/min/1.73 m^2^ [21].

### DNA extraction

Approximately 2–3 ml of venous blood was collected by a sterile venipuncture using a sterile EDTA vacutainer. The blood samples were put in EDTA anticoagulant tubes, stored in the refrigerator at −20°C until use. Genomic DNA was extracted from EDTA anticoagulated whole blood using QIAamp DNA Mini Kit (QIAGEN; Hilden, Germany) according to the manufacturer’s protocol. The extracted DNA was dissolved in TE buffer (10 mM Tris, 1 mM EDTA; pH = 7.8), quantified by measuring the absorbance at 260 nm, and then stored at −20°C for genotyping.

### SNP genotyping

The primer was designed by Generunner 6.2.07 β-software and synthesized by Beijing Liuhe Huada Gene Company. The genotype was completed by the PCR-restriction fragment length polymorphism (PCR-RFLP). The region of the *CYP3A4* (G/A rs4646437) encompassing SNP was amplified using the TaqMan® 5’ allelic discrimination technique for amplifying and detecting specific polymorphisms in purified genomic DNA samples. The sense sequence was (5-CAAAGAATCCCAATTTTGGCAGAG-3), while the antisense sequence was (5-TCAGTCCCTGGGGTGAGAG-3). The TaqMan® MGB probes/extension primers were designed to detect the allele 1 sequence and to detect the allele 2 sequence. The reaction system included 12 μl of 2 × Es Taq Master Mix, 2 μl forward and reverse, and primer with 10 μl mol/l, 2 μl DNA templates, and 9 μl. The PCR program consisted of the following steps: initial denaturation step at 94°C for 2 min, followed by 30 cycles: denaturation at 98°C for 10 s, annealing at 61°C for 30 s, extension at 72°C for 30 s, and extension again at 72°C for 2 min, and stored at 4°C. The PCR product was incubated overnight at 37°C with restriction enzyme. Then the mixtures were electrophoresed and visualized. The expected fragment were: 196 bp in GG; 196, 158, and 38 bp in GA; and 158 and 38 bp in AA.

### Statistical analysis

We used the SPSS 24.0 platform to complete all analysis. The quantitative data was expressed by using means ± standard deviation, and independent *t* test was used for comparison between the case and control group. The qualitative variables were expressed using count and percent, and χ^2^ test was used for comparison between two groups. The Hardy–Weinberg equilibrium (HWE) was tested to evaluate whether the control group could represent the whole population by a goodness-of-fit χ^2^ test. The rude and adjusted ORs with 95% CIs were calculated to assess the relationship between *CYP3A4* gene and hypertension. *P*<0.05 was considered as a significant level.

## Results

### General characteristic between case group and control group

The comparisons of general characteristics between controls and cases were presented in Tables 1 and 2. For demographic data, there were 160 males and 359 females in the control group and 168 males and 282 females in the case group, respectively. Groups significantly differ by the number of participants in gender subgroups (*P*=0.033). The mean ages were 46.8 and 45.7 in the case and control group, respectively. The smoking rate of control group was 13.5 and 16.0% in the case group. The rate of alcohol consumption was 36.9% in the control and was 43.8% in the case group. No significant differences were observed for age, smoking, and alcohol consumption (*P*>0.05). We also investigated the time of exercise/week. As we can see from the Table 1, the rate of exercise twice a week was almost equal in the control group and case group (14.4 and 14.7%), and there was no significant difference between the two groups (*P*=0.103). The salt intake based on self report indicated that moderate intake rate was also equal between two groups (52.9 vs 53.1%, *P*=0.925). There was also no significant difference in the rate of education level and marriage status between case group and control group (*P*=0.999, *P*=0.937).

**Table 1:** Comparisons of general characteristics between controls and cases

Variables	Control (*n*=520)	Cases (*n*=450)	t/χ^2^	*P*-value
Sex			4.554	0.033
Male	160 (44.6%)	168 (37.3%)		
Female	359 (55.4%)	282 (62.7%)		
Age (y)	46.8±13.3	45.7±12.5	1.646	0.187
Smoking			1.244	0.265
Yes	70 (13.5%)	72 (16.0%)		
No	450 (86.5%)	378 (84.0%)		
Alcohol consumption			4.719	0.030
No	192 (36.9%)	197 (43.8%)		
Yes	328 (63.1%)	253 (56.3%)		
Exercise/week			7.702	0.103
None	270 (51.9%)	206 (45.8%)		
One	98 (18.8%)	117 (26.0%)		
Twice	75 (14.4%)	66 (14.7%)		
More	77 (14.8%)	61 (13.5%)		
Salt intake			0.155	0.925
Low	184 (35.4%)	155 (34.4%)		
Moderate	275 (52.9%)	239 (53.1%)		
High	61 (11.7%)	49 (10.9%)		
Education			0.106	0.999
None or primary	145 (27.9%)	128 (28.4%)		
Middle	172 (33.1%)	150 (33.3%)		
High	134 (25.8%)	112 (24.9%)		
College	69 (13.2%)	60 (13.3%)		
Marriage			0.131	0.937
Unmarried	180 (34.8%)	159 (35.3%)		
Married	322 (62.0%)	277 (61.6%)		
Others	18 (3.2%)	14 (3.1%)		

**Table 2:** Comparisons of biochemical indexes between controls and cases

Variables	Control (*n*=520)	Cases (*n*=450)	t	*P-value*
BMI, kg/m^2^	26.9 ± 3.5	26.7 ± 3.6	0.876	0.381
FBG, mg/dl	5.6 ± 0.6	5.8 ± 0.7	−4.791	0.000
TC, mg/dl	5.1 ± 0.9	5.0 ± 0.9	1.725	0.085
TG, mg/dl	1.8 ± 0.5	1.9 ± 0.5	−3.106	0.002
HDL-C, mg/dl	1.1 ± 0.2	1.1 ± 0.2	0.000	0.999
LDL-C, mg/dl	3.2 ± 0.8	3.2 ± 0.9	1.904	0.057
BUN, mg/dl	4.7 ± 1.1	4.9 ± 1.2	−2.707	0.007
Creatinine, mg/dl	67.6 ± 13.0	73.3 ± 12.7	−6.883	0.000
Uric acid, mg/dl	350.9 ± 49.1	351.7 ± 46.4	−0.259	0.795
eGFR, ml/min	72.3 ± 19.6	70.5 ± 21.0	1.380	0.168
ALT, U/l	21.9 ± 28.0	21.1 ± 21.2	0.496	0.620
AST, U/l	25.2 ± 10.8	24.5 ± 9.0	1.087	0.277
RBC, ×10^12^/l	4.9 ± 0.4	4.9 ± 0.6	−309	0.757
WBC, ×10^9^/l	6.5 ± 1.8	6.6 ± 1.7	−0.885	0.376
Hb, g/l	138.5 ± 15.0	141.1 ± 14.1	−2.661	0.008
Plt, ×10^9^/l	246.2 ± 54.2	234.2 ± 57.5	1.454	0.147
RDW, %	12.6 ± 0.7	12.9 ± 0.9	−5.831	0.000
rs4646437			18.169	0.000
GG	303 (58.3%)	246 (54.7%)		
GA	176 (33.8%)	129 (28.7%)		
AA	41 (7.9%)	75 (16.7%)		

The Table 2 presented the comparisons of biochemical index between controls and cases. The BMI was 26.9 and 26.7 in the two groups, respectively. There were no significant differences in BMI. The FBG level of the case group was significantly higher than that in the control group (5.8 vs 5.6, *P*=0.000). The TG level was also higher in the case group than that in the control group (*P*=0.002). However, no significant differences were observed in TC, HDL-C, and LDL-C level between two groups (*P*=0.085, *P*=0.999, *P*=0.057). The BUN level (4.9 vs 4.7, *P*=0.007) and creatinine level (73.3 vs 67.6, *P*=0.000) were higher in the case group than that in the control group. We also estimated the eGFR level between two groups. There were no significant differences (70.5 vs 72.3, *P*=0.168) between case group and control group. No significant differences were also observed in ALT and AST. Our results also indicated that the RDW and Hb levels were higher in the case group than that in the control group. The RDW was 12.9 and 12.6, respectively and the Hb of two groups was 141.1 and 138.5 g/l, respectively. The details of all results are presented in Table 2.

### Association of *CYP3A4*/ rs4646437 gene polymorphism with hypertension

The genotype distribution of *CYP3A4* rs4646437 were presented in the Table 2. The GG, GA, and AA genotype were 303 (58.3%), 176 (33.8%), and 41 (7.9%) in the control group and were 246 (54.7%), 129 (28.7%), and 75 (16.7%) in the case group. As we can see, the AA genotype frequency was significantly higher in the case group than that in the control group (16.7 vs 7.9%, *χ^2^*=18.169, *P*=0.000). We explored the relationship between rs4646473 gene polymorphism and hypertension using the following model: dominant model (GA + AA vs GG), recessive model (AA vs GG + GA), additive model (GA vs GG, AA vs GA), and allele genetic model (A vs G). For dominant model, the GA/AA genotype did not increase the risk of hypertension compared with GG genotype (*OR*=1.16, 95% CI: 0.90–1.49, *P*=0.259). Compared with GG/GA genotype, the AA genotype also increased the risk of hypertension (*OR*=2.34, 95% CI: 1.56–3.50, *P*=0.000). For additive model, the AA genotype was significantly associated with GG genotype (*OR*=2.25, 95% CI: 1.49–3.42, *P*=0.000). The same results were found for AA vs GA (*OR*=2.50, 95% CI: 1.60–3.89, *P*=0.000). For the allele genotype, the A allele frequency was significantly higher in the case group than that in the control group (31.0 vs 24.8%, *P*=0.002). The A-allele of *CYP3A4* rs4646437 was associated with an increased risk for hypertension (*OR*=1.36, 95% CI: 1.12–1.66, *P*=0.002). All results are presented in Table 3.

**Table 3:** Association between gene models of CYP3A4 gene locus and hypertension

SNP	Model	Control		Case		χ^2^	*P-value*	OR	95% CI
		n	%	n	%				
rs4646437	A vs G	258/782	24.8/75.2	279/621	31.0/69.0	9.242	0.002	1.36	1.12–1.66
	AA vs GA	41/176	18.9/81.1	75/129	36.8/63.2	16.823	0.000	2.50	1.60–3.89
	GA vs GG	176/303	36.7/63.3	129/246	34.4/65.6	0.503	0.478	0.90	0.68–1.20
	AA vs GG	41/303	11.9/88.1	75/246	23.3/76.7	15.106	0.000	2.25	1.49–3.42
	GA + AA vs GG	217/303	41.7/58.3	204/246	45.3/54.7	1.275	0.259	1.16	0.90–1.49
	AA vs GG + GA	41/479	7.8/92.1	75/375	16.7/83.3	17.671	0.000	2.34	1.56–3.50

### Multivariable logistic regression analysis

To exclude the potential confounding factors, we performed the multivariable analyses by adjusting the variables with significant difference in the univariate analysis including sex, alcohol consumption, FBG, TG, BUN, creatinine, Hb, and RDW. Our results indicated that AA (*OR*=2.23, 95% CI: 1.47–3.39, *P*=0.000) was significantly associated with the risk of hypertension compared with GG genotype, and GA genotype was not related to hypertension risk compared with GG. Besides, we also confirmed that the FBG and BUN levels will increase the risk of hypertension (*OR*=2.33, 95% CI: 1.31–4.14, *P*=0.004 and *OR*=1.04, 95% CI: 1.02–1.06, *P*<0.001). All results are presented in Table 4.

**Table 4:** Multivariable logistics regression analysis between rs4646437 and hypertension

Variables	β	*SE*	Wald*χ^2^*	*P*-value	OR	95% CI
FBG	0.844	0.293	8.276	0.004	2.33	1.31–4.14
BUN	0.038	0.011	13.202	<0.001	1.04	1.02–1.06
Male	1.814	0.299	36.394	<0.001	6.14	3.42–11.01
GG					1.00	
GA	−0.063	0.145	0.186	0.667	0.94	0.71–1.25
AA	0.801	0.214	14.037	0.000	2.23	1.47–3.39
Constant	−11.430	2.095	29.773	<0.001		

## Discussion

In the present study, we found that the A allele genotype of *CYP3A4* rs4646437 can increase the risk of hypertension compared with G allele genotype. The multivariable results indicated that the significant association between rs4646437 and hypertension still existed. Hence, the CYP3A4 rs4646437 may be associated with occurrence of hypertension. Having considered the drug metabolism function of *CYP3A4*, the rs4646437 may be a potential treatment target for hypertension with A allele mutation.

It was widely acknowledged that hypertension is a chronic disease determined by genetic factors and environment factors. A lot of studies have reported the hypertension was related to genetic, ethnic and areas. It was reported that many drug metabolism-related gene also have gene mutations [22,23]. The association between gene variation and drug metabolism provided new insights for the treatment of diseases like hypertension [24]. CYP3A4 is one of the subfamilies of CYP450, mainly secreted by liver and small intestine. It was previously reported that CYP3A4 is mainly associated with drug metabolism. Considerable interindividual heterogeneity exists in the expression of CYP3A4 both in and out of the liver, which is to a large part genetically determined. The *CYP3A4* gene is located on chromosome 7qq22.1, in the vicinity of the *CYP3A5, CYP3A4*, and *CYP3A7* genes. These CYP3As have similar function [25]. While over 28 SNPs have been identified in the *CYP3A4* gene, it has been found that this does not translate into significant interindividual variability *in vivo*. It can be supposed that this may be due to the induction of CYP3A4 on exposure to substrates. *CYP3A4* alleles which have been reported to have minimal function compared with wild-type include CYP3A4*6 (an A17776 insertion) and CYP3A4*17 (F189S) [26]. Both of these SNPs led to decreased catalytic activity with certain ligands, including testosterone and nifedipine in comparison to wild-type metabolism. Variability in CYP3A4 function can be determined by the erythromycin breath test (ERMBT). The ERMBT estimates *in vivo* CYP3A4 activity by measuring the radiolabeled carbon dioxide exhaled after an intravenous dose of (14C-N-methyl)-erythromycin [27].

A study from Chinese population indicated that the *CYP3A4* gene polymorphism was associated with hypertension. However, this is a study with small sample size (69 cases and 66 control), which make the results unstable [28]. The present study has larger sample size and provides stronger evidences. Currently, the potential biological mechanism by which the *CYP3A4* gene affects the progression of hypertension still remains unclear. However, we may get some clues from its congener CYP3A5. The CYP3A5 has similar function and property as the *CYP3A4*. It has been suggested that cortisol may play a critical role in the regulation of blood pressure and the development of hypertension. Animal experiments have suggested that excessive intracranial conversion of cortisol to 6β-hydroxy corticosterone via increased renal CYP3A5 activity, which is regulated by the CYP3A5 gene, could enhance postrenal proximal tubular sodium re-absorption, thus resulting in elevated blood pressure levels [29]. However, CYP3A could also stabilize the expression of corticosterone-induced active sodium transport in kidney cells by mineralocorticoid receptor-mediated mechanisms, which could then lead to decreased BP. This may be the underlying mechanism by which the *CYP3A4* allele results in a lower SBP. This mechanism needs to be further confirmed in vivo and vitro experiments. A number of antihypertensive drugs have been shown to be CYP3A substrates. Among them are the widely prescribed drugs enalapril, amlodipine, and losartan [30]. Note that CYP3A5 and CYP3A4 share many antihypertensive drug substrates in common. CYP3A5 genotypes were associated with the response to verapamil, an antihypertensive drug in health subjects [31]. These genotypes were also associated with the blood pressure response to verapamil in blacks and Hispanics and with blood pressure response to an ACE inhibitor in subjects of African descent [32]. This may partly explain the potential mechanism of CYP3A4 associated with hypertension.

The present study has several limitations. First, the present study design was based on case–control design, the causal relationship was limited. Second, some study population may took some other medicines but did not report the history of usage. Furthermore, the CYP3A4 was associated with drug metabolism, which may have some underestimated or overestimated effect on the present results. However, at least we excluded these hypertensions with the history of antihypertension drug. Third, some data collection was based on self report, such as salt intake, smoking, and alcohol consumption. Some accurate measurements may be more appropriate. Finally, the present study explored the relationship between *CYP3A4* and hypertension using epidemiology, and the molecular mechanisms need to be confirmed.

In conclusion, we found that the *CYP3A4* gene rs4646473 polymorphism was related to the risk of hypertension in the Chinese population, and this effect could be confirmed by multivariable analyses. Future studies should focus on the mechanisms and the interaction between the *CYP3A4* gene and other genes and environmental factors. Future studies will allow a better estimation of the role of variants located within the CYP3A5 genes in blood pressure control in the general population, including interethnic differences. With the increasing availability of genome-wide data, gene–gene interactions throughout the entire genome will be considered. More research will be conducted to better characterize endogenous CYP3A5 substrates and to understand how these may interact with exogenous substrates. CYP3A5 will influence the choice of antihypertensive drugs.

## Abbreviations

ACEI: angiotensin-converting enzyme
ALT: alanine amino ramsferase
AST: aspartate amino transferase
BMI: body mass index
BP: blood pressure
BUN: blood urea nitrogen
CI: confidence interval
CYP: cytochrome P450
eGFR: estimated glomerular filtration rate
EH: essential hypertension
ERMBT: erythromycin breath test
FBG: fasting blood glucose
GFR: glomerular filtration rate
HDL-C: high-density lipoprotein
LDL-C: low-density lipoprotein
OR: odds ratio
Plt: platelet count
RBC: red blood cell
SBP: systolic blood pressure
SNP: single nucleotide polymorphism
TC: total cholesterol
TE: Trisedta
TG: triglyceride
WBC: white blood cell

## References

[1] Burnier M., Oparil S., Narkiewicz K. and Kjeldsen S.E. (2018) New 2017 American Heart Association and American College of Cardiology guideline for hypertension in the adults: major paradigm shifts, but will they help to fight against the hypertension disease burden? Blood Press. 27, 62–65 10.1080/08037051.2018.1430504 29447001

[2] Bundy J.D. and He J. (2016) Hypertension and related cardiovascular disease burden in China. Ann. Glob. Health 82, 227–233 10.1016/j.aogh.2016.02.002 27372527

[3] Kim H.C., Jeon Y.W. and Heo S.T. (2018) Global Impact of the 2017 American College of Cardiology/American Heart Association Hypertension guidelines. Circulation 138, 2312–2314 10.1161/CIRCULATIONAHA.118.036312 30571579

[4] Wang J., Zhang L., Wang F., Liu L. and Wang H. (2014) Prevalence, awareness, treatment, and control of hypertension in China: results from a national survey. Am. J. Hypertens. 27, 1355–1361 10.1093/ajh/hpu053 24698853 PMC4263934

[5] Okeahialam B.N., Alonge B.A., Pam S.D. and Puepet F.H. (2011) Carotid intima media thickness as a measure of cardiovascular disease burden in nigerian africans with hypertension and diabetes mellitus. Int. J. Vasc. Med. 2011, 327171 21748020 10.1155/2011/327171 PMC3124892

[6] Qamar A. and Braunwald E. (2018) Treatment of hypertension: addressing a global health problem. JAMA 320, 1751–1752 10.1001/jama.2018.16579 30398610

[7] Fisher N. and Curfman G. (2018) Hypertension-A Public Health Challenge of Global proportions. JAMA 320, 1757–1759 10.1001/jama.2018.16760 30398584

[8] Huo Y., Li J., Qin X., Huang Y., Wang X., Gottesman R.F. (2015) Efficacy of folic acid therapy in primary prevention of stroke among adults with hypertension in China: the CSPPT randomized clinical trial. JAMA 313, 1325–1335 10.1001/jama.2015.2274 25771069

[9] Cooper R.S., Kaufman J.S. and Bovet P. (2017) Global burden of disease attributable to hypertension. JAMA 317, 2017–2018 10.1001/jama.2017.4213 28510671

[10] Song C.K., Martinez J.A., Kailasam M.T., Dao T.T., Wong C.M., Parmer R.J. (2000) Renal kallikrein excretion: role of ethnicity, gender, environment, and genetic risk of hypertension. J. Hum. Hypertens. 14, 461–468 10.1038/sj.jhh.1001047 10918552

[11] Zhang X., Zhao H., Zhang J., Han D., Zheng Y., Guo X. (2015) Gene environment interaction of GALNT2 and APOE gene with hypertension in the Chinese Han Population. Biomed. Mater. Eng. 26, S1977–S1983 26405973 10.3233/BME-151501

[12] Rodriguez-Iturbe B. and Johnson R.J. (2019) Genetic polymorphisms in hypertension: are we missing the immune connection? Am. J. Hypertens. 32, 113–122 10.1093/ajh/hpy168 30418477

[13] Rhodes C.J., Batai K., Bleda M., Haimel M., Southgate L., Germain M. (2018) Genetic determinants of risk in pulmonary arterial hypertension: international genome-wide association studies and meta-analysis. Lancet Respir. Med. 7, 227–238 10.1016/S2213-2600(18)0409-0 30527956 PMC6391516

[14] Gu S., Kumar R., Lee M.H., Mickael C. and Graham B.B. (2019) Common genetic variants in pulmonary arterial hypertension. Lancet Respir. Med. 7, 190–191 10.1016/S2213-2600(18)30448-X 30527955 PMC6768546

[15] Liu Y.T., Hao H.P., Liu C.X., Wang G.J. and Xie H.G. (2007) Drugs as CYP3A probes, inducers, and inhibitors. Drug Metab. Rev. 39, 699–721 10.1080/03602530701690374 18058330

[16] Hannemann F., Bichet A., Ewen K.M. and Bernhardt R. (2007) Cytochrome P450 systems–biological variations of electron transport chains. Biochim. Biophys. Acta 1770, 330–344 10.1016/j.bbagen.2006.07.017 16978787

[17] Ghodke-Puranik Y.A. and Lamba J.K. (2017) Pharmacogenomics. In Innovative Approaches in Drug Discovery (Patwardhan B. and Chaguturu R., eds)., pp. 195–234, Academic Press, Boston 10.1016/B978-0-12-801814-9.00007-6

[18] Zanger U.M. and Schwab M. (2013) Cytochrome P450 enzymes in drug metabolism: regulation of gene expression, enzyme activities, and impact of genetic variation. Pharmacol. Ther. 138, 103–141 10.1016/j.pharmthera.2012.12.007 23333322

[19] Diekstra M.H., Belaustegui A., Swen J.J., Boven E., Castellano D., Gelderblom H. (2017) Sunitinib-induced hypertension in CYP3A4 rs4646437 A-allele carriers with metastatic renal cell carcinoma. Pharmacogenomics J. 17, 42–46 10.1038/tpj.2015.100 26810136

[20] Hesketh T. and Zhou X. (2017) Hypertension in China: the gap between policy and practice. Lancet 390, 2529–2530 10.1016/S0140-6736(17)32743-5 29102085

[21] McFarlane P., Cherney D., Gilbert R.E. and Senior P. (2018) Chronic kidney disease in diabetes. Can. J. Diabetes 42, S201–S209 10.1016/j.jcjd.2017.11.004 29650098

[22] Brown J.T., Bishop J.R., Sangkuhl K., Nurmi E.L., Mueller D.J., Dinh J.C. (2019) Clinical pharmacogenetics implementation consortium (CPIC) guideline for CYP2D6 genotype and atomoxetine therapy. Clin. Pharmacol. Ther. 93, 402–408 10.1002/cpt.1409 30801677 PMC6612570

[23] Altman R.B. (2007) PharmGKB: a logical home for knowledge relating genotype to drug response phenotype. Nat. Genet. 39, 426 10.1038/ng0407-426 17392795 PMC3203536

[24] Turner S.T., Bailey K.R., Fridley B.L., Chapman A.B., Schwartz G.L., Chai H.S. (2008) Genomic association analysis suggests chromosome 12 locus influencing antihypertensive response to thiazide diuretic. Hypertension 52, 359–365 10.1161/HYPERTENSIONAHA.107.104273 18591461 PMC2692710

[25] Bochud M., Bovet P., Burnier M. and Eap C.B. (2009) CYP3A5 and ABCB1 genes and hypertension. Pharmacogenomics 10, 477–487 10.2217/14622416.10.3.477 19290795

[26] Lee S.J. and Goldstein J.A. (2005) Functionally defective or altered CYP3A4 and CYP3A5 single nucleotide polymorphisms and their detection with genotyping tests. Pharmacogenomics 6, 357–371 10.1517/14622416.6.4.357 16004554

[27] Watkins P.B. (1994) Noninvasive tests of CYP3A enzymes. Pharmacogenetics 4, 171–184 10.1097/00008571-199408000-00001 7987401

[28] Zuo L., Fang F., Yang J., Sui X.F., Zhang Z.G. and Lei L.L. (2015) Analysis on CYP3A4*1G gene polymorphism in health and hypertension population from Jiamusi area. Heilongjiang Med. Pharm. 38, 7–9

[29] Morris D.J., Latif S.A., Rokaw M.D., Watlington C.O. and Johnson J.P. (1998) A second enzyme protecting mineralocorticoid receptors from glucocorticoid occupancy. Am. J. Physiol. 274, C1245–C1252 10.1152/ajpcell.1998.274.5.C1245 9612211

[30] Siest G., Jeannesson E. and Visvikis-Siest S. (2007) Enzymes and pharmacogenetics of cardiovascular drugs. Clin. Chim. Acta 381, 26–31 10.1016/j.cca.2007.02.014 17362901

[31] Langaee T.Y., Gong Y., Yarandi H.N., Katz D.A., Cooper-DeHoff R.M., Pepine C.J. (2007) Association of CYP3A5 polymorphisms with hypertension and antihypertensive response to verapamil. Clin. Pharmacol. Ther. 81, 386–391 10.1038/sj.clpt.6100090 17339868

[32] Jin Y., Wang Y.H., Miao J., Li L., Kovacs R.J., Marunde R. (2007) Cytochrome P450 3A5 genotype is associated with verapamil response in healthy subjects. Clin. Pharmacol. Ther. 82, 579–585 10.1038/sj.clpt.6100208 17443134

